# Architecture of the Cutaneous Autonomic Nervous System

**DOI:** 10.3389/fneur.2019.00970

**Published:** 2019-09-10

**Authors:** Patrick Glatte, Sylvia J. Buchmann, Mido Max Hijazi, Ben Min-Woo Illigens, Timo Siepmann

**Affiliations:** ^1^Department of Neurology, University Hospital Carl Gustav Carus, Technische Universität Dresden, Dresden, Germany; ^2^Department of Neurosurgery, University Hospital Carl Gustav Carus, Technische Universität Dresden, Dresden, Germany; ^3^Department of Neurology, Beth Israel Deaconess Medical Center, Harvard Medical School, Boston, MA, United States

**Keywords:** C-fiber, punch skin biopsy, axon-reflex, Parkinson's disease, diabetes, skin, autonomic (vegetative) nervous system, autonomic neuropathy

## Abstract

The human skin is a highly specialized organ for receiving sensory information but also to preserve the body's homeostasis. These functions are mediated by cutaneous small nerve fibers which display a complex anatomical architecture and are commonly classified into cutaneous A-beta, A-delta and C-fibers based on their diameter, myelinization, and velocity of conduction of action potentials. Knowledge on structure and function of these nerve fibers is relevant as they are selectively targeted by various autonomic neuropathies such as diabetic neuropathy or Parkinson's disease. Functional integrity of autonomic skin nerve fibers can be assessed by quantitative analysis of cutaneous responses to local pharmacological induction of axon reflex responses which result in dilation of cutaneous vessels, sweating, or piloerection depending on the agent used to stimulate this neurogenic response. Sensory fibers can be assessed using quantitative sensory test. Complementing these functional assessments, immunohistochemical staining of superficial skin biopsies allow analysis of structural integrity of cutaneous nerve fibers, a technique which has gained attention due to its capacity of detecting pathogenic depositions of alpha-synuclein in patients with Parkinson's disease. Here, we reviewed the current literature on the anatomy and functional pathways of the cutaneous autonomic nervous system as well as diagnostic techniques to assess its functional and structural integrity.

## Introduction

Small fiber neuropathy is a condition which leads to impaired functional integrity of unmyelinated autonomic or somatic small nerve fibers. This condition affects approximately 53 per 100.000 people and the most common etiologies are diabetes, neurodegenerative diseases, and complex regional pain syndrome due to trauma and paraneoplastic syndromes (1). Autonomic small fiber neuropathy has been associated with increased morbidity and mortality in patients with diabetes or cardiovascular disease (1–3). Only a few diagnostic techniques are available to assess peripheral small fiber neuropathy. The most commonly used technique in terms of quantitative and qualitative analyses is the skin biopsy, including different immunohistochemical staining methods which are either not fiber specific or allow specific analysis of cholinergic or adrenergic nerve fibers. Assessment of functional integrity of these nerve fibers can be performed using Laser doppler flowmetry (LDF), two-dimensional Laser doppler imaging (LDI) as well as axon-reflex based tests of sudomotor and pilomotor function such as the quantitative sudomotor axon reflex test (QSART) and the quantitative pilomotor axon reflex test (QPART) (4–8). Modern treatments are chiefly directed to the pathophysiological mechanisms causing selective damage to small nerve fibers. When causative therapy is not available personalized symptomatic treatment regimens can substantially improve quality of life. To optimize treatment and identify new therapeutic targets, it is essential to acknowledge the architecture and physiology of the peripheral small nerve fibers. An easily accessible model of peripheral small nerve fibers is the study of the cutaneous nervous system, which is also impaired by small fiber neuropathies and due to its involvement in some neurodegenerative disorders has been referred to a “window into brain pathology” (9).

The cutaneous innervation consists of both autonomic (predominantly sympathetic but in face also parasympathetic) and sensory nerve fibers (10, 11). These nerve fibers derive from perikarya located in the dorsal root and sympathetic ganglia. The outcoming nerve fibers can be subclassified due to their different conduction velocity, properties of perception and signaling and affiliation into “afferent-sensory” or/and “efferent-autonomic” as well as into Aβ, Aδ, and C-fibers. The cutaneous nervous system is excitable to different stimuli including exogenous as well as endogenous stimuli. Exogenous stimuli comprise mechanical forces, chemicals, ultraviolet light rays, electrical & thermal stimuli (12). Endogenous stimuli are more complex, and it has to be distinguished between physiological and pathophysiological stimuli, which derive from cells of the neuro-immuno-endocrine system found in the skin (13). This review focusses on autonomic C and Aδ-fibers, as they are selectively targeted by various neuropathies and play a crucial regulatory role in physiological functions of the skin, such as thermoregulation, wound healing, and hydration. We aimed to provide an update on the architecture and diagnostic assessments of the autonomic cutaneous nervous system.

## Search Strategy

A narrative review of the current literature on the architecture of the autonomic cutaneous nervous system was undertaken. Literature search was performed by using Medline and the Cochrane Library (Cochrane Database of Systematic Reviews, Cochrane Central Register of Controlled Trials (CENTRAL) and Cochrane Methodology Register. Additionally, the reference lists of the selected studies were also screened. No restrictions on language or date were applied during the literature research. Literature search was performed between 01/09/2018 and 23/06/2019 The search strategy included the search term “small fibers” in combination with each of the following terms;“skin,” “autonomic” “sympathetic,” “parasympathetic,” “anatomy,” “myelinated,” “unmyelinated,” “C-fibers,” “A-delta fibers”, “cutaneous,” “pilomotor,” “sudomotor,” “vasomotor” using the Boolean operators “AND” as well as “OR” and their combinations.

## Functional Classification of Cutaneous Nerve Fibers

Axons of the peripheral nervous system can be subdivided based on their specific action potential conduction velocities (CV) into nerve fibers of the class A, B, and C, whereas A fibers exhibit the fastest conduction velocity and C-fibers the slowest. They also show different specifications, functions and distributions in the human body (11). Sensory class A fibers arise from pseudounipolar sensory neurons of the dorsal root ganglia, which pass on their sensory input to neurons in the dorsal horn of the spinal cord. They mix immediately after exiting the ganglia with the nerve bundles of motor neurons from the anterior horn of the spinal cord to supply sensory and motor innervation to the periphery of the body (12, 14–17). Type β and δ belong to the nerve fibers giving sensory innervation for the skin. Type β fibers are thick, highly myelinated, rapid conducting fibers with mechanoreceptive properties (12, 15–18). Type Aδ fibers are thinly myelinated nerve fibers with narrower diameter and slower conduction velocity. Type Aδ fibers allow sensory input for mechanical and heat nociception and non-noxious cold thermal stimuli (12, 14, 16, 18, 19).

Class B and efferent C-fibers are nerve fibers of the sympathetic nervous system, which is a part of the autonomic nervous system. The sympathetic nervous system supplies the body with efferent innervation, which adjusts the body's organ function to its environment, for example in the skin by regulating the body temperature via eccrine sweat gland stimulation (sudomotor), vasoconstriction (vasomotor) or arrector pili stimulation (pilomotor). The sympathetic fiber origin is the lateral gray horn of the spinal cord's segments C/8Th1 – L3. General visceral efferent preganglionic neurons send out thinly myelinated white rami communicantes made of class B-preganglionic-fibers. These class B-fibers provide cholinergic innervation to the postganglionic neurons in the paravertebral ganglions that possess nicotinic acetylcholine receptors for stimulation (8). Long postganglionic class C-fibers exit via gray rami communicantes to join major peripheral nerves to provide vasoconstrictive adrenergic innervation to blood vessels and the arrector pili muscles but paradoxically also cholinergic innervation for the stimulation of eccrine sweat glands and vasodilation of blood vessels (7, 8, 20–25). Besides the sympathetic C-fibers, studies have shown that other Class C-fibers derive from sensory neurons of the dorsal root ganglia to provide sensory innervation to the skin. These sensory C-fibers, alike A-fibers, can be classified into C-HTMR and C-LTMR fibers (12, 16, 17). Both exhibit different properties and react to different stimuli. Studies have shown that C-HTMR fibers allow the perception of noxious mechanical and thermal, non-noxious warm/cold thermal stimuli and chemical stimuli (19, 26–29). Recent analyses have shown that C-LTMR (C-tactile)-fibers percept pleasant mechanical stimuli in glabrous and hairy skin (30, 31). It has also been shown that C-tactile-fibers possess the ability to modulate pain perception (32). Type Aδ and C fibers are referred to as polymodal because of their ability to sense various different stimuli. All class C-fibers are unmyelinated axons in groups (Remak bundles) of 2–8, wrapped by the cytoplasm of a centrally located single Schwann cell. Because of the missing myelination and narrow axon diameter, the C-fibers' conduction velocity is the lowest among all fiber classes (<2 m/s) (12, 17, 18, 26, 33, 34). The diameter of a C-fiber can reach from 0.2 to 1.4 μm whereas axons in the epidermal layer (0.5–1.4 μm) have a larger diameter than axons in the dermal layer (0.2–0.6 μm) (35). Table 1 compares the most important anatomical and (electro)-physiological features of the different cutaneous nerve fibers.

**Table 1:** Comparison of fiber characteristics.

**Class**	**Type**	**Myelination**	**Diameter μm**	**CV $\frac{\text{m}}{\text{s}}$**	**$\frac{F}{A}$ or $\frac{F}{mm}$**	**Skin type**	**Location**	**Associated organ**	**Stimuli**	**References**
A-β	SAI-LTMR	Thick	6–12 μm	35–75 $\frac{m}{s}$	$\frac{100\;F}{cm^{2}}$	Hairy & glabrous	Stratum basale epidermis, guard hair	Merkels disc, guard hair	Mechanical fine touch	(12, 15–18, 36, 37)
	SAII-LTMR	Thick			$\frac{10\;F}{cm^{2}}$	Glabrous	Dermis	Ruffini corpuscle	Mechanical stretch	
	RAI-LTMR	Thick			$\frac{159\;F}{cm^{2}}$	Glabrous	Dermal papillae	Meissner corpuscle	Mechanical deformation	
	RAII-LTMR	Thick			$\frac{20\;F}{cm^{2}}$	Glabrous	Deep dermis	Pacinian corpuscle	Mechanical vibration	
A-δ	HTMR	Thin	1–5 μm	5–30 $\frac{m}{s}$	$\frac{7,2-10,9\;F}{mm}$	Hairy, glabrous	Epidermis>dermis	Free nerve endings	Noxious heat & mechanical	(12, 14, 16–18, 36, 37)
	LTMR	Thin			male: $\frac{7,2-10,9F}{mm}$ female: $\frac{6,7-13,5F}{mm}$	Hairy	Dermis, epidermis	Free nerve endings, zigzag + auchene hair	Mechanical, non-noxious cold	
B		Thin	3 μm	3–15 $\frac{m}{s}$	-	-	White rami communicantes	Paravertebral ganglion		
C	C-sympathetic efferent	Unmyelinated	0.2–1.4 μm	<2 $\frac{m}{s}$	PNF leg + thigh, VIP, DβH mean (SD): 52.3–63.7 (12.4–18.2) $\frac{F}{mm}\;$ SNF: 40.8–51.3(11.8–13.2%) PID	Hairy, glabrous	Dermis	Eccrine sweat glands, vessels, arrector pili, free nerve endings	Cholinergic agents, catecholamines	(8, 36–42)
	HTMR afferent	Unmyelinated			male: $\frac{7,2-10,9F}{mm}$ female: $\frac{6,7-13,5\;F}{mm}\;$	Hairy, glabrous	Epidermis>dermis	Free nerve endings	Noxious -heat, cold, mechanical	(12, 16–18, 30, 36, 37)
	LTMR/C-tactile afferent	Unmyelinated			male: $\frac{7,2-10,9F}{mm}$ female: $\frac{7,2-10,9\;F}{mm}\;$	Hairy, glabrous	Epidermis>dermis	Free nerve endings, zigzag & auchene hair	Pleasant mechanical, non-noxious heat, rapid cooling	

*Table summarizing physiological and anatomical characteristics of different types of nerve fibers. SA, Slow Adapting; RA, Rapid Adapting; LTMR, Low Threshold Mechanoreceptors; HTMR, High Threshold Mechanoreceptors; CV, Conduction Velocity; F, Fiber; A, Area; PNF, Pilomotor Nerve Fiber; VIP, Vasoactive Intestinal Peptide; DβH, Dopamine beta Hydroxylase; SNF, Sudomotor Nerve Fiber; PID, Percent Intercept Density*.

## General Anatomy of Cutaneous Autonomic Nerve Fibers and Associated Organs

**Figure 1:**
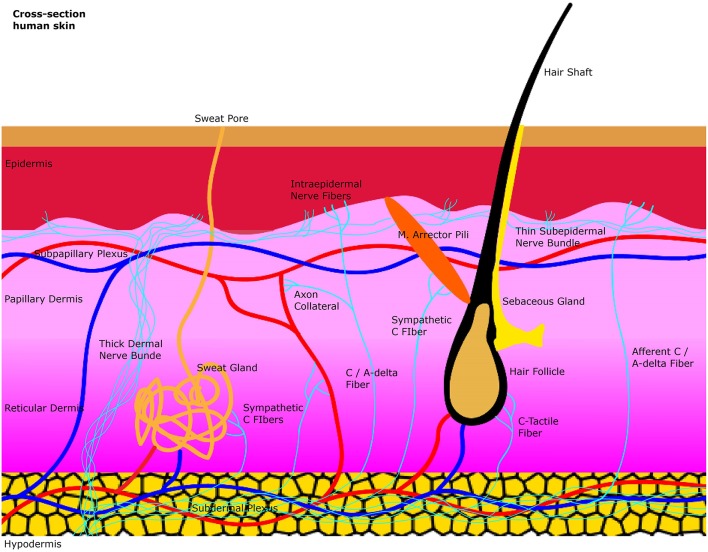
A simplified illustration of the general anatomy of the skin with the focus on autonomic nerve fibers and their innervated organs. Sweat glands, blood vessels and the arrector pili muscle are innervated by sympathetic C-fibers in the dermis. Afferent intraepidermal nerve fibers of the class C and Aδ are found in the epidermis as free nerve endings. Axon collaterals of these afferent fibers also supply blood vessels with efferent antidromic control. Small sensory fibers branch off from thicker dermal nerve bundles to create thinner subepidermal nerve bundles that innervate the epidermis.

The skin is composed superficially of the epidermis which is an epithelial layer. More profound lies the dermis which is a connective tissue layer. The junction between dermis and epidermis is folded due to dermal papillae invaginating into epidermal ridges. Beneath the dermis lies the hypodermis which is subcutaneous tissue composed mainly of fat cells. The dermis can be subdivided into the outermost papillary layer and deeper reticular layer. This division is supported by the anatomical arrangement of two major arteriovenous plexuses. At the interface between hypodermis and dermis lies the subdermal plexus which is connected through arteriovenous shunts to the subpapillary plexus between reticular and papillary dermis. The subpapillary plexus sends branches to the dermal papillae. The thermoregulatory control of these Vessels relies on input of the accompanying postganglionic unmyelinated C-fibers of the autonomic sympathetic nervous system. Epidermal derivatives located in the dermis like hair follicles and their associated arrector Pili smooth muscles as well as eccrine sweat glands are also effector organs of postganglionic sympathetic C-fibers. Polymodal sensory afferents in the dermis include unmyelinated C-fibers and thinly myelinated Aδ fibers which are nociceptors, mechanoceptors, and thermoceptors. These fibers supply hair follicles and epidermis through myelin free nerve endings but also send collaterals to other organs like blood vessels to generate sympathetic mediated axon reflexes. Epidermal/dermal free nerve endings are also called intraepidermal/dermal nerve fibers. The densities of these epidermal/dermal nerve fibers are a major diagnostical target for small fiber neuropathy (Figure 1).

Currently, only few normative datasets of dermal nerve fiber densities (DNFD) associated with cutaneous organs controlled by autonomic nerve fibers are available compared to intraepidermal nerve fiber densities (IENFD) for unmyelinated nerve fibers. Available datasets were created by means of punch skin biopsies from the distal calf above the lateral malleus, distal and proximal thigh. They show an intraepidermal nerve fiber density that reduces with age as well as course of neuropathy and which differs among sexes. In addition, the values of examined IENFD vary due to the dependency on different staining and microscopy techniques, creating an inhomogeneity between publications. According to a worldwide normative reference study the median normative IENFD (ages combined) ranged in males from 7.2 to 10.9 $\frac{F}{mm}$ and in females from 6.7 to 13.5 $\frac{F}{mm}$ (F = Fibers) (35, 43, 44). A study of Nolano and Colleagues quantified pilomotor nerves to compare the fiber density those of diabetic patients (38). In the group of healthy control subjects, mean(SD) pilomotor nerve fiber density in the leg and thigh visualized by immunhistochemistry using antibodies against substances like vasoactive intestinal peptide (VIP) and dopamine-β-hydroxylase (DβH) ranged from 52.3 to 63.7 (12.4–18.2) $\frac{F}{mm}$ (38). In another study, sudomotor nerve fibers have been assessed using the manual quantitation method. A grid of circles was placed over the sweat gland image of interest. Sweat gland nerve fibers crossing the circles within the grid were counted in a three-dimensional stepping pattern and set in relation with the total number of circles within the area of the grid in. This enabled a percent area counting method. The sweat gland nerve fiber density (SGNFD) was therefore expressed in percent intercept density. In control subjects, the mean(SD) SGNFD at the distal leg was 40.8 ± 12.8%, at the distal thigh 46.6 ± 13.2%, and at the proximal thigh 51.3 ± 11.8% (39). To date, there is no large normative dataset for autonomic nerve fiber densities around the cutaneous effector organs. There is a need for closing this knowledge gap in order to use the skin biopsy technique for the diagnosis of autonomic neuropathies in individual patients.

## Sudomotor, Vasomotor, And Pilomotor Nerve Fibers As Potential Diagnostic Targets

**Figure 2:**
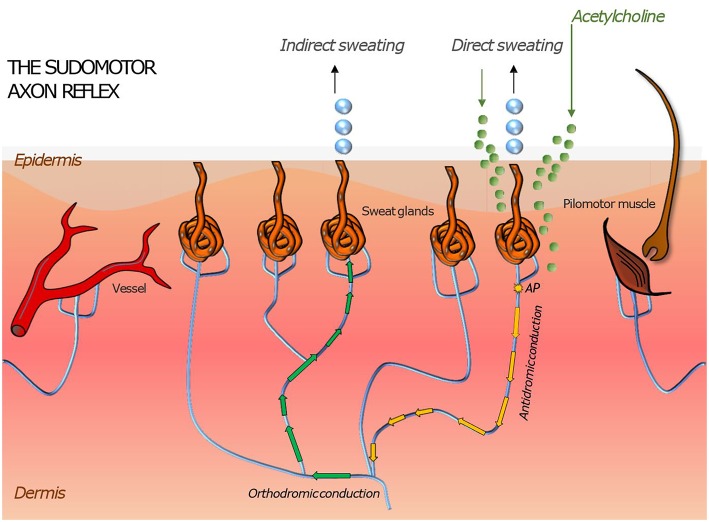
Illustration of skin organs innervated by the autonomic nervous system with an axon reflex mediated in sudomotor nerve fibers by iontophoretic application of acetylcholine to the skin. Following a direct sweat response in the area of acetylcholine application, an action potential travels antidromically and then orthodromically to a neighboring population of sweat glands where it evokes “indirect” sweating in a skin region adjacent to the region of iontophoresis. Similar responses can be induced in pilomotor and vasomotor fibers. Their magnitude is a surrogate measure of functional integrity of the autonomic nerve fiber mediating the axon reflex.

Small fiber neuropathy can impair the functioning of the thermoregulation, which is a crucial regulatory and physiological process in human organism. Thermoreceptors such as Aδ and C-fibers provide sensory information from the human skin, which is processed in the hypothalamus, the regulatory center responsible for monitoring the body temperature. The hypothalamus combines thermal information of the skin, internal organs, preoptic anterior hypothalamus, and non-thermal information related to thermoregulation (8, 45, 46). Via the sympathetic pathway, the eccrine sweat production and vasodilation in the skin can be stimulated to reduce the body temperature. Similar effects can be evoked peripherally through the sudomotor, vasomotor and pilomotor axon reflex. The axon reflex has been firstly described in 1900 by Langley. However, recent studies show a wide range of heterogeneity of axon reflexes regarding the type of applied agonists as well as the type of stimulated fibers. The sudomotor axon reflex can be evoked by a local iontophoresis of cholinergic agonists such as acetylcholine. These agents bind on muscarinic receptors of the sweat gland to stimulate a direct sweat production locally and bind on nicotinic cholinergic receptors on postganglionic sympathetic C-fiber terminals to activate an antidromic impulse along the postganglionic sympathetic C-fiber. At the branching points of this fiber, it changes to an orthodromic impulse stimulating neighboring eccrine sweat glands through an indirect axon reflex mediated cholinergic agent release (8, 22, 23, 45, 47). The clinically applied quantitative sudomotor axon reflex test is a tool used to detect autonomic neuropathy (Figure 2).

Recent studies have also shown, that non-sympathetic afferent C and Aδ fibers can elicit an axon reflex. To elicit the vasomotor axon reflex iontophoresis of cholinergic agents is used to cause a direct vasodilatory response and a depolarization of non-sympathetic sensory nociceptive Aδ and C-fibers and sympathetic C-fibers. In contrast to the sudomotor axon reflex, the nerve impulse travels orthodromically to a branching point. From this branching point the potential is conducted antidromically toward a terminal bouton at a blood vessel. There, the release of vasoactive neuropeptides causes an indirect axon reflex mediated vasodilatory response. On the other hand, vasoconstriction can be induced by a local application of adrenergic agents (6, 20, 48–50).

Malfunctions of the vasomotor axon reflexes in the skin due to an autonomic neuropathy can be detected by tests such as laser Doppler flowmetry, laser Doppler imaging. In research studies laser doppler flowmetry has been shown reliable in the detection of differences in vasomotor function between patients with autonomic neuropathy and controls. However, due to high intersubject variability, use of the technique is limited in the clinical diagnostic setting. More advanced techniques such as laser Doppler imaging have displayed lower variability in some smaller studies (48). However the technique's validity has not yet been tested in large populations of patients with autonomic neuropathy. Therefore, both techniques remain on an experimental level at this stage and are mainly limited to specialized centers.

In the pilomotor axon reflex, the direct arrector pili muscle erection (also referred to as pilomotor erection, goose bump) and orthodromic sympathetic C-fiber nerve impulse can be generated by iontophoresis of the adrenergic agent phenylephrine. At branching points the impulse travels antidromically to induce an indirect axon reflex mediated activation of neighboring pilomotor muscles. Since LTMR sensory C-fibers are known to be related to Zigzag and Auchene hair follicles, there may be a pilomotor axon reflex pathway via sensory C-fibers. In research, quantitative pilomotor axon reflex (QPART) testing has been introduced as a diagnostic method in the detection of peripheral small fiber neuropathy but these experimental observations have not yet been translated into clinical practice (7, 24, 51). Therefore, standard testing for small fiber neuropathy in the skin currently relies on the punch skin biopsy. This structural measure can be complemented by functional tests of sensory function (quantitative sensory testing) and sudomotor function (QSART), the latter being however limited by high technical demands and low sensitivity for general small fiber loss. A discussion on these cutaneous autonomic tests is given in sections Structural Measures of Cutaneous Autonomic Neuropathy and Disorders of the Cutaneous Autonomic Nervous System and Their Assessment.

The way how axon reflexes result in effector response upon conduction of the action potential to terminal nerve endings has not been fully elucidated. However, several neuropeptides have been identified which apparently contribute to inducing these responses upon neurogenic stimulation. Neuropeptides involved in inducing axon reflex mediated responses are shown in Table 2.

**Table 2:** Neuropeptides & their function in the skin.

**Neuropeptide**	**Releaser**	**Receptor**	**Organ**	**Function**
CGRP	Mainly sensory C-fibers	CGRP-receptor	Blood vessels, eccrine sweat glands, hair follicles, meissner corpuscle, free nerve endings	Vasodilation
SP	Mainly sensory c-fibers	TACR1-receptor	Blood vessels	Vasodilation, pain, inflammation
VIP	Sympathetic & sensory c-fibers	VIPR 1/2	Blood vessels, eccrine sweat glands, hair follicles	Vasodilation, eccrine sweat gland stimulation
DβH	sympathetic C- fibers		Blood vessels, m. arrector pili	Enzyme for dopamine oxidation to noradrenalin
Tyrosine hydroxylase	Sweat gland neuroendocrine cells		Sweat gland neuroendocrine cells	Enzyme transforming L-tyrosine to L-DOPA
Neuropeptide Y	Sympathetic C- fibers	GPCRs Y1/2/4/6	Blood vessels	Vasoconstriction
Catecholamines	Sympathetic C- fibers	Adrenergic-α-1- receptor	Blood vessels, m. arrector pili	Vasoconstriction, m. arrector pili erection
Acetylcholine	Sympathetic C- fibers	Nicotinic acetylcholine receptors, muscarinic receptors	Blood vessels, sensory & sympathetic C- fibers, eccrine sweat glands	Vasodilation, axon-reflex, eccrine sweat gland stimulation
Capsaicin	None	TRPV1	Sensory C & A-δ fibers	Vasodilation
Menthol	None	TRPM8	Sensory C & A-δ fibers	Vasoconstriction

*Table of neuropeptides relevant to function and structure of small nerve fibers, highlighting their effects on the effector organs. CGRP, Calcitonin Gene Related Peptide; SP, Substance P; VIP, Vasoactive Intestinal Peptide; DβH, Dopamine beta Hydroxylase; TACR1, Tachykinin Receptor 1; VIPR $\frac{1}{2}$, Vasoactive Intestinal Peptide Receptor 1/2; GPCR, G Protein Coupled Receptors Y1/2/4/6; TRPV1, Transient Receptor Potential Subfamily V Member 1; TRPM8, Transient Receptor Potential Cation Channel Subfamily M Member 8) (10, 13, 41, 52–58)*.

## Structural Measures of Cutaneous Autonomic Neuropathy

**Figure 3:**
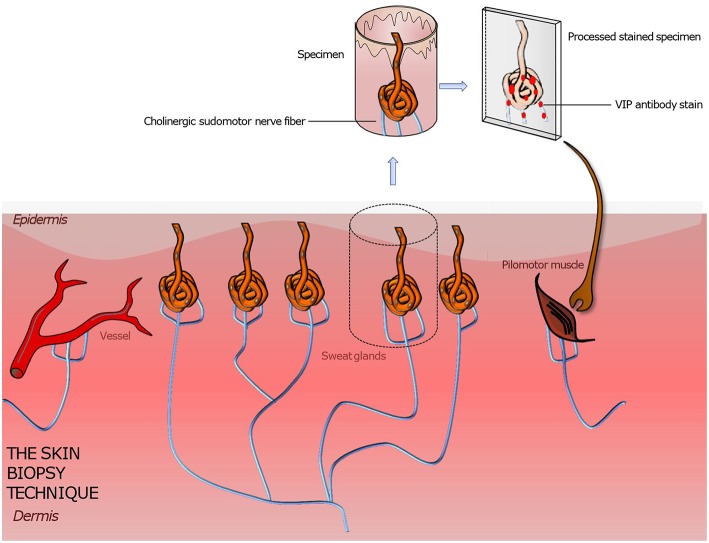
Illustration of a punch skin biopsy on eccrine sweat glands to quantify the cholinergic sudomotor nerve fibers. The specimen is fixed, sectioned, and stained with antibodies for PGP 9,5 (the pan axonal marker), tyrosine hydroxylase (a sweat gland neuroendocrine cell marker), and VIP (a marker for sympathetic nerve fibers) to highlight the sought-after tissue. Further various quantitation methods are applied to assess the sweat gland nerve fiber density. Based on this technique pilomotor and vasomotor autonomic nerve fibers can be quantified by using suitable staining methods. A comparison of the determined nerve fiber density to those of normative datasets gives information about the functionality and condition of the autonomic nervous system innervating skin organs.

The development and research on punch skin biopsies provided a tool to gain insight into the structure of small nerve fibers. Unlike the nerve biopsy the punch biopsy obtains a (usually 3 × 3 mm) specimen with low risk of adverse effects (Figure 3). A subcutaneous lidocaine injection is performed prior to the procedure to numb the skin area. The area undergoing the biopsy usually does not require any stiches or steri stripes to heal. The biopsy can be done by any clinician trained in the technique, but the processing of the biopsy requires special expertise of the pathology lab. Due to the axon length dependency of peripheral neuropathy, symptoms first make a symmetrical ascent from the terminals of the longest nerves, which are in the leg. Reason is the high metabolic rate in long axons which makes them vulnerable for metabolic disorders like diabetes (59–61). Therefore, standard biopsy sites are the distal calf, distal thigh, proximal thigh or generally the site affected by symptoms. After the processing of the sample, different immunohistochemical staining methods can be applied to visualize the material of interest (4, 35, 43). To generally visualize all nerve fibers in the sample, the pan-axonal marker, protein gene peptide 9,5 antibody is applied (52). This is the most common technique used to assess the intraepidermal/dermal nerve fiber density. To further distinguish sensory nerve fibers from autonomic nerve fibers and cholinergic autonomic from adrenergic autonomic nerve fibers additional immunostaining methods have been developed for different antigens or substances located in the specific nerve fibers.

To quantify the adrenergic innervation around the arrector pili muscle DβH staining is applied. On the other hand, the VIP marker can be used to assess cholinergic innervation around the sweat gland. Other antibodies including substance P, CGRP or neuropeptide Y are also used in order to highlight different autonomic nerve fibers and their distributions as summarized in detail in Table 2 (4, 10, 42, 62).

Microneurography studies have been shown to capture efferent sympathetic nerve activity. Tungsten needle electrodes are inserted into a nerve to capture the activity of multi or single nerve fibers. The procedure is minimally invasive and therefore doesn't require any anesthetics so the patient can be awake and interact with the physician. To take the tip of the electrode to contact the particular nerve fiber the electrode has to pass through the layers of a nerve. Since the nerve consists of multiple nerve fascicles with mixed nerve fibers each wrapped by a connective tissue layer (epi-, peri-, endo-neurium) the searching process can be time consuming and requires highly skilled physicians which limits the procedure's use as standard diagnostical method in clinical routine.

Axon-reflex tests such as QSART, QPART, LDF, and LDI have been designed to indirectly evaluate functional integrity of autonomic small fibers. In contrast to these, the punch skin biopsy immediately visualizes the affected nerves on a structural level. By counting the IENFD/DNFD and scanning the fibers' morphology i.e., for axonal swellings, both qualitative and quantitative assessment of the integrity of these fibers can be undertaken (35, 43). Limitations of the punch skin biopsy technique include a lack of sufficient normative datasets regarding the autonomic nerve fiber densities. To date, there is no comprehensive and externally valid normative dataset for autonomic nerve fiber densities around the cutaneous effector organs. It seems necessary to close this knowledge gap in order to be able to use the punch skin biopsy purposefully for the diagnosis of neuropathies of the autonomic cutaneous nervous system. Physicians are also limited to IENFD data from the distal calf, thigh and proximal thigh, making it difficult to make diagnosis of other areas with physically visible symptoms and especially of dermal located effector organs affected by an autonomic neuropathy. In a comparison study of three methods for the quantification of SGNFD an automated counting method for SGNFD showed a high degree of inter- and intra-reviewer reliability, the technique correlated well with intra-epidermal nerve fiber density, clinical neuropathy exam scores and an unbiased confocal microscopy stereologic analysis of sweat gland nerve fiber density. Furthermore, it proved to be less labor intensive and easily reproducible requiring only 1 min per image for analysis compared to 15 min per sweat gland with the manual counting method (40). Nevertheless, there exists a wide heterogeneity of nerve fiber densities between gender, age and ethnicity. Further limitations may include the accessibility to laboratories performing the processing of the obtained samples.

## Disorders of the Cutaneous Autonomic Nervous System and Their Assessment

Patients with Parkinson's disease suffer from various symptoms beside the motor function restriction. Those affecting the cutaneous autonomic nervous system include a dysfunction in thermoregulation due to an impairment of the vasomotor and sudomotor activity in two thirds of the patients. The coarse reason for this interference are the accompanying pathological consequences of the peripheral small fiber neuropathy (2, 4). Although the pathophysiology is not yet understood sufficiently, recent studies provide evidence that accumulation of molecularly misfolded alpha-synuclein may be a major factor in the development of Parkinson's disease (24, 51, 63–66). A study using immunohistochemical imaging of punch skin biopsies from Parkinson's patients showed that alpha-synuclein was increased and protein gene product 9,5 (PGP 9,5) was reduced in autonomic adrenergic and cholinergic C-fibers, indicating an overall loss of nerve fibers and deposition of alpha-synuclein in the remaining fibers. Whereas alpha-synuclein was not found in intraepidermal nociceptive nerve fibers according (64). This could be one of the reasons why vasomotor axon reflex testing via the LDF may not be an adequate diagnostical method for patients with Parkinson disease, since it relies on the vasomotor axon reflex of nociceptive Aδ and C-fibers (5, 24, 48, 51). Conversely, in another investigation alpha- synuclein has been found in somatosensory subepidermal nerve fibers, which include nociceptive A-δ and C- fibers in patients with Parkinson's disease (65). Nevertheless, the ratio between alpha-synuclein and PGP 9,5 generated from punch skin biopsies seems to be a reliable disease marker of Parkinson's Disease. Pilomotor function has been found impaired in patients with Parkinson's disease by evaluating the pilomotor mediated axon reflex using the Quantitative Pilomotor Axon Reflex Test (51). These findings were in line with biopsy based analyses showing strong impairment of structural integrity of pilomotor nerve fibers even in early stages of the disease (64).

Other diseases affecting the cutaneous autonomic nervous system include pre-diabetes and diabetes, traumas and reconstructions of peripheral nerves. In fact, diabetic neuropathy is the most frequent for autonomic neuropathies. The Consensus Panel on Diabetic Neuropathy reported a prevalence of diabetes related cardiovascular autonomic neuropathy of 20% up to 60% with increasing age and diabetes duration in patients suffering from type 1 and 2 diabetes (67, 68). Diabetic Autonomic Neuropathy (DAN) must be taken serious due to the high mortality rate of 16–50% within 5 years in diabetes related cardiovascular autonomic neuropathy patients (68, 69). The pathophysiology of DAN has not yet been fully understood. Hyperglycemia results in various metabolic, immune, neurotrophic, and vascular changes. These abnormal conditions cause either direct degeneration of neurons, axons and Schwann cells or indirect degeneration due to a progressive damage to the vasa nervorum. Symptoms of DAN can occur in single or multiple organ systems including the cardiovascular, gastrointestinal, genitourinary, respiratory, neuroendocrine, pupillomotor, and neurovascular system. Impairment in the neurovascular system affects the autonomic cutaneous nervous system, resulting in sweating abnormalities (anhidrosis, hyperhidrosis, gustatory sweating), unusual pilomotor and vasomotor control. This pathology leads to a dysfunction of the body's thermoregulation (38, 68, 70). Cardiovascular tests, which include an ECG, blood pressure and heart rate are considered the gold standard diagnostical methods for evaluating DAN because of their non-invasiveness, sensitivity, and reproducibility. Tests of the autonomic cutaneous nervous system in DAN have been applied less by default (68, 70, 71). Nevertheless, techniques such as QSART, QPART, LDF and LDI as well as the punch skin biopsy technique may serve as additional diagnostical methods to assess a DAN via the autonomic cutaneous nervous system. Taking into account the wide range of affected systems, it seems reasonable to contemplate cutaneous autonomic tests alongside the unilateral cardiovascular tests (38, 72). A cross-sectional study published in 2016 with 103 obese participants and 53 lean controls reported a prevalence of 11.1% for polyneuropathy including small fiber neuropathy in the obese participants with normoglycemia compared to 25.5% in obese participants with type 2 diabetes and 3.8% in lean controls. This finding underscores the widespread importance in acquiring new knowledge about the cutaneous nervous system (73).

Furthermore, small autonomic nerve fibers are selectively targeted by various systemic diseases neuropathies related to amyloid, autoimmune autonomic neuropathies comprising those caused by a paraneoplastic syndrome, hereditary autonomic neuropathies, autonomic neuropathies resulting from infectious diseases as well as toxic autonomic neuropathies (3).

## Conclusion

The autonomic cutaneous nervous system has shown to be an increasing focus of interest, particularly in the last decade. This can be explained by the functional relevance of the autonomic skin nerves playing a major role in physiological functions such as thermoregulation as well as by the observation that these fibers seem to be selectively targeted in early stages of neurodegenerative diseases such as Parkinson's disease. Techniques to assess both functional and structural integrity of skin nerve fibers have been developed and technically improved, overcoming initial issues of technical demands and intersubject variability. The punch skin biopsy technique is still limited by the lack normative datasets. This, however, does not surpass the advantages of this newly developed technique, allowing for qualitative and quantitative assessment of structural integrity of skin nerves and, thereby, complementing functional small fiber tests such as axon reflex tests. With improvement in assessing the neural skin architecture in humans on a structural and functional level, our anatomical and physiological understanding of this important system evolves, introducing the opportunity to identify novel diagnostic and therapeutic targets of small fiber neuropathy.

## Author Contributions

PG has drafted the first version of the manuscript. SB, MH, BI, and TS have made substantial contributions by reviewing the manuscript for intellectual content, language, and design.

### Conflict of Interest Statement

The authors declare that the research was conducted in the absence of any commercial or financial relationships that could be construed as a potential conflict of interest.

## References

[1] Peters MJ Bakkers M Merkies IS Hoeijmakers JG van Raak EP Faber CG. Incidence and prevalence of small-fiber neuropathy: a survey in the Netherlands. Neurology. (2013) 81:1356–60. 10.1212/WNL.0b013e3182a8236e 23997150

[2] Chan AC Wilder-Smith EP. Small fiber neuropathy: getting bigger! Muscle Nerve. (2016) 53:671–82. 10.1002/mus.25082 26872938

[3] Freeman R. Autonomic peripheral neuropathy. Lancet. (2005) 365:1259–70. 10.1016/S0140-6736(05)74815-7 15811460

[4] Donadio V Incensi A Giannoccaro MP Cortelli P Stasi VD Pizza F. Peripheral autonomic neuropathy: diagnostic contribution of skin biopsy. J Neuropathol Exp Neurol. (2012) 71:1000–8. 10.1097/NEN.0b013e3182729fdc 23037327

[5] Frerichs KU Feuerstein GZ. Laser-Doppler flowmetry. A review of its application for measuring cerebral and spinal cord blood flow. Mol Chem Neuropathol. (1990) 12:55–70. 10.1007/BF03160057 2278606

[6] Illigens BMW Siepmann T Roofeh J Gibbons CH. Laser-doppler imaging in the detection of peripheral neuropathy. Auton Neurosci. (2013) 177:286–90. 10.1016/j.autneu.2013.06.006 23850386 PMC3778150

[7] Siepmann T Gibbons CH Illigens BM Lafo JA Brown CM Freeman R. Quantitative Pilomotor axon-reflex test – a novel test of pilomotor function. Arch Neurol. (2012) 69:1488–92. 10.1001/archneurol.2012.1092 22868966 PMC3563419

[8] Illigens BMW Gibbons CH. Sweat testing to evaluate autonomic function. Clin Auton Res. (2009) 19:79–87. 10.1007/s10286-008-0506-8 18989618 PMC3046462

[9] Doppler K Ebert S Uceyler N Trenkwalder C Ebentheuer J Volkmann J. Cutaneous neuropathy in Parkinson's disease: a window into brain pathology. Acta Neuropathol. (2014) 128:99–109. 10.1007/s00401-014-1284-0 24788821 PMC4059960

[10] Nolano M Provitera V Caporaso G Stancanelli A Leandri M Biasiotta A. Cutaneous innervation of the human face as assessed by skin biopsy. J Anat. (2013) 222:161–9. 10.1111/joa.12001 23078075 PMC3632221

[11] Siemionow M Gharb BB Rampazzo A. The face as a sensory organ. Plastic Reconstr Surg. (2011) 127:652–62. 10.1097/PRS.0b013e3181fed6fd 21285770

[12] Abraira VE Ginty DD. The sensory neurons of touch. Neuron. (2013) 79:618–39. 10.1016/j.neuron.2013.07.051 23972592 PMC3811145

[13] Roosterman D Goerge T Schneider SW Bunnett NW Steinhoff M. Neuronal control of skin function: the skin as a neuroimmunoendocrine organ. Physiol Rev. (2006) 86:1309–79. 10.1152/physrev.00026.2005 17015491

[14] Djouhri L. Aδ-fiber low threshold mechanoreceptors innervating mammalian hairy skin: a review of their receptive, electrophysiological and cytochemical properties in relation to Aδ-fiber high threshold mechanoreceptors. Neurosci Biobehav Rev. (2016) 61:225–38. 10.1016/j.neubiorev.2015.12.009 26746589

[15] Fleming MS Luo W. The anatomy, function, and development of mammalian Aβ low-threshold mechanoreceptors. Front Biol. (2013) 8:408–20. 10.1007/s11515-013-1271-1 24376457 PMC3873732

[16] Li L Rutlin M Abraira Victoria E Cassidy C Kus L Gong S. The functional organization of cutaneous low-threshold mechanosensory neurons. Cell. (2011) 147:1615–27. 10.1016/j.cell.2011.11.027 22196735 PMC3262167

[17] Roudaut Y Lonigro A Coste B Hao J Delmas P Crest M. Touch sense: Functional organization and molecular determinants of mechanosensitive receptors. Channels. (2012) 6:234–45. 10.4161/chan.22213 23146937 PMC3508902

[18] Fang X McMullan S Lawson SN Djouhri L. Electrophysiological differences between nociceptive and non-nociceptive dorsal root ganglion neurones in the rat *in vivo*. J Physiol. (2005) 565:927–43. 10.1113/jphysiol.2005.086199 15831536 PMC1464557

[19] Lawson SN Phenotype and function of somatic primary afferent nociceptive neurones with C-, Aδ- or Aα/β-Fibres. Exp Physiol. (2002) 87:239–44. 10.1113/eph8702350 29345433

[20] Al-Horani RA Mohammad M. The contribution of noradrenergic nerves to the vasoconstrictor response during local cooling of leg and forearm skin in humans. Gen Physiol Biophys. (2018) 37:33–40. 10.4149/gpb_2017021 29424350

[21] Vetrugno R Liguori R Cortelli P Montagna P. Sympathetic skin response: basic mechanisms and clinical applications. Clin Auton Res. (2003) 13:256–70. 10.1007/s10286-003-0107-5 12955550

[22] Jenkinson DM Montgomery I Elder HY. Studies on the nature of the peripheral sudomotor control mechanism. J Anat. (1978) 125:625–39. 640964 PMC1235629

[23] Low PA Caskey PE Tuck RR Fealey RD Dyck PJ. Quantitative sudomotor axon reflex test in normal and neuropathic subjects. Ann Neurol. (1983) 14:573–80. 10.1002/ana.410140513 6316835

[24] Siepmann T Frenz E Penzlin AI Goelz S Zago W Friehs I. Pilomotor function is impaired in patients with Parkinson's disease: a study of the adrenergic axon-reflex response and autonomic functions. Parkinsonism Relat Disord. (2016) 31:129–34. 10.1016/j.parkreldis.2016.08.001 27569843

[25] Sternini C. Organization of the peripheral nervous system: autonomic and sensory ganglia. J Investig Dermatol Symp Proc. (1997) 2:1–7. 10.1038/jidsymp.1997.2 9487007

[26] Van Hees J Gybels J. C nociceptor activity in human nerve during painful and non painful skin stimulation. J Neurol Neurosurg Psychiatry. (1981) 44:600–7. 7288447 10.1136/jnnp.44.7.600 PMC491064

[27] Alvarez FJ Fyffe REW. Nociceptors for the 21st Century. Curr Rev Pain. (2000) 4:451–8. 10.1007/s11916-000-0069-4 11060591

[28] Schmelz M Schmidt R Weidner C Hilliges M Torebjork EH Handwerker H. Chemical response pattern of different classes of C-nociceptors to pruritogens and algogens. J Neurophysiol. (2003) 89:2441–8. 10.1152/jn.01139.2002 12611975

[29] Campero M Bostock H. Unmyelinated afferents in human skin and their responsiveness to low temperature. Neurosci Lett. (2010) 470:188–92. 10.1016/j.neulet.2009.06.089 19576956

[30] Liljencrantz J Olausson H. Tactile C fibers and their contributions to pleasant sensations and to tactile allodynia. Front Behav Neurosci. (2014) 8:37. 10.3389/fnbeh.2014.00037 24639633 PMC3944476

[31] Triscoli C Olausson H Sailer U Ignell H Croy I CT-optimized skin stroking delivered by hand or robot is comparable. Front Behav Neurosci. (2013) 7:208 10.3389/fnbeh.2013.00208 24391564 PMC3866892

[32] Habig K Schänzer A Schirner W Lautenschläger G Dassinger B Olausson H. Low threshold unmyelinated mechanoafferents can modulate pain. BMC Neurol. (2017) 17:184. 10.1186/s12883-017-0963-6 28915853 PMC5603029

[33] Schmelz M Schmidt R Bickel A Torebjörk HE Handwerker HO. Innervation territories of single sympathetic C fibers in human skin. J Neurophysiol. (1998) 79:1653–60. 10.1152/jn.1998.79.4.1653 9535936

[34] Ertekin C Ertekin N Karcioglu M. Conduction velocity along human nociceptive reflex afferent nerve fibres. J Neurol Neurosurg Psychiatry. (1975) 38:959–65. 10.1136/jnnp.38.10.959 1202167 PMC492130

[35] Collongues N Samama B Schmidt-Mutter C Chamard-Witkowski L Debouverie M Chanson J-B. Quantitative and qualitative normative dataset for intraepidermal nerve fibers using skin biopsy. PLoS ONE. (2018) 13:e0191614. 10.1371/journal.pone.0191614 29370274 PMC5784950

[36] Obi T Takatsu M Yamazaki K Kuroda R Terada T Mizoguchi K. Conduction velocities of Adelta-fibers and C-fibers in human peripheral nerves and spinal cord after CO2 laser stimulation. J Clin Neurophysiol. (2007) 24:294–7. 10.1097/WNP.0b013e318038f45f 17545835

[37] Li CL Bak A. Excitability characteristics of the A- and C-fibers in a peripheral nerve. Exp Neurol. (1976) 50:67–79. 10.1016/0014-4886(76)90236-3 1248547

[38] Nolano M Provitera V Caporaso G Stancanelli A Vitale DF Santoro L. Quantification of pilomotor nerves: a new tool to evaluate autonomic involvement in diabetes. Neurology. (2010) 75:1089–97. 10.1212/WNL.0b013e3181f39cf4 20855852

[39] Gibbons CH Illigens BMW Wang N Freeman R. Quantification of sweat gland innervation: a clinical–pathologic correlation. Neurology. (2009) 72:1479–86. 10.1212/WNL.0b013e3181a2e8b8 19398703 PMC2677479

[40] Gibbons CH Illigens BMW Wang N Freeman R Quantification of sudomotor innervation: a comparison of 3 methods. Muscle Nerve. (2010) 42:112–9. 10.1002/mus.21626 20544913 PMC3048308

[41] Bjorklund H Dalsgaard CJ Jonsson CE Hermansson A. Sensory and autonomic innervation of non-hairy and hairy human skin. An immunohistochemical study. Cell Tissue Res. (1986) 243:51–7. 10.1007/BF00221851 2417723

[42] Wang N Gibbons CH. Chapter 30: Skin biopsies in the assessment of the autonomic nervous system. In: Buijs RM, Swaab DF, editors. Handbook of Clinical Neurology Vol. 117. Elsevier (2013). p. 371–8. 10.1016/B978-0-444-53491-0.00030-4 24095140

[43] Pereira MP Mühl S Pogatzki-Zahn EM Agelopoulos K Ständer S. Intraepidermal nerve fiber density: diagnostic and therapeutic relevance in the management of chronic pruritus: a review. Dermatol Therap. (2016) 6:509–17. 10.1007/s13555-016-0146-1 27730494 PMC5120635

[44] Lauria G Bakkers M Schmitz C Lombardi R Penza P Devigili G. Intraepidermal nerve fiber density at the distal leg: a worldwide normative reference study. J Periph Nervous Syst. (2010) 15:202–7. 10.1111/j.1529-8027.2010.00271.x 21040142

[45] Low PA. Evaluation of sudomotor function. Clin Neurophysiol. (2004) 115:1506–13. 10.1016/j.clinph.2004.01.023 15203051

[46] Guttmann L Silver J Wyndham CH. Thermoregulation in spinal man. J Physiol. (1958) 142:406–19. 10.1113/jphysiol.1958.sp006026 13576444 PMC1356752

[47] Namer B Bickel A Kramer H Birklein F Schmelz M. Chemically and electrically induced sweating and flare reaction. Auton Neurosci. (2004) 114:72–82. 10.1016/j.autneu.2004.06.007 15331047

[48] Kubasch ML Kubasch AS Torres Pacheco J Buchmann SJ Illigens BM-W Barlinn K. Laser doppler assessment of vasomotor axon reflex responsiveness to evaluate neurovascular function. Front Neurol. (2017) 8:370. 10.3389/fneur.2017.00370 28855885 PMC5557735

[49] Ochoa J Yarnitsky D Marchettini P Dotson R Cline M. Interactions between sympathetic vasoconstrictor outflow and C nociceptor-induced antidromic vasodilatation. Pain. (1993) 54:191–6. 10.1016/0304-3959(93)90208-7 8233533

[50] Berghoff M Kathpal M Kilo S Hilz MJ Freeman R. Vascular and neural mechanisms of ACh-mediated vasodilation in the forearm cutaneous microcirculation. J Appl Physiol. (2002) 92:780–8. 10.1152/japplphysiol.01167.2000 11796692

[51] Siepmann T Pintér A Buchmann SJ Stibal L Arndt M Kubasch AS. Cutaneous Autonomic Pilomotor Testing to Unveil the Role of Neuropathy Progression in Early Parkinson's Disease (CAPTURE PD): protocol for a multicenter study. Front Neurol. (2017) 8:212. 10.3389/fneur.2017.00212 28603514 PMC5445122

[52] Dalsgaard C-J Rydh M Hægerstrand A. Cutaneous innervation in man visualized with protein gene product 9.5 (PGP 9.5) antibodies. Histochemistry. (1989) 92:385–90. 10.1007/BF00492495 2531128

[53] Bossaller C Reither K Hehlert-Friedrich C Auch-Schwelk W Graf K Grafe M. *In vivo* measurement of endothelium-dependent vasodilation with substance P in man. Herz. (1992) 17:284–90. 1282120

[54] Anand P Bloom SR McGregor GP. Topical capsaicin pretreatment inhibits axon reflex vasodilatation caused by somatostatin and vasoactive intestinal polypeptide in human skin. Br J Pharmacol. (1983) 78:665–9. 10.1111/j.1476-5381.1983.tb09418.x 6133573 PMC2044739

[55] Wong BJ Tublitz NJ Minson CT. Neurokinin-1 receptor desensitization to consecutive microdialysis infusions of substance P in human skin. J Physiol. (2005) 568:1047–56. 10.1113/jphysiol.2005.095372 16123103 PMC1464169

[56] Lotti T Hautmann G Panconesi E. Neuropeptides in skin. J Am Academy Dermatol. (1995) 33:482–96. 10.1016/0190-9622(95)91395-5 7657872

[57] Laverdet B Danigo A Girard D Laurent M Demiot C Alexis D. Skin innervation: important roles during normal and pathological cutaneous repair. Histol Histopathol. (2015). 30:875–92. 25799052 10.14670/HH-11-610

[58] Ashrafi M Baguneid M Bayat A. The role of neuromediators and innervation in cutaneous wound healing. Acta Derm Venereol. (2016) 96:587–94. 10.2340/00015555-2321 26676806

[59] Baloh RH. Mitochondrial dynamics and peripheral neuropathy. Neuroscientist. (2008) 14:12–8. 10.1177/1073858407307354 17911220

[60] Misra UK Kalita J Nair PP. Diagnostic approach to peripheral neuropathy. Ann Indian Acad Neurol. (2008) 11:89–97. 10.4103/0972-2327.41875 19893645 PMC2771953

[61] Chung T Prasad K Lloyd TE. Peripheral neuropathy: clinical and electrophysiological considerations. Neuroimaging Clin N Am. (2014) 24:49–65. 10.1016/j.nic.2013.03.023 24210312 PMC4329247

[62] Myers MI Peltier AC Li J. Evaluating dermal myelinated nerve fibers in skin biopsy. Muscle Nerve. (2013) 47:1–11. 10.1002/mus.23510 23192899 PMC3528842

[63] Siepmann T Penzlin AI Illigens BM-W Reichmann H. Should skin biopsies be performed in patients suspected of having Parkinson's Disease? Parkinson's Dis. (2017) 2017:6064974. 10.1155/2017/6064974 29214093 PMC5682910

[64] Wang N Gibbons CH Lafo J Freeman R. α-Synuclein in cutaneous autonomic nerves. Neurology. (2013) 81:1604–10. 10.1212/WNL.0b013e3182a9f449 24089386 PMC3806913

[65] Doppler K Jentschke H-M Schulmeyer L Vadasz D Janzen A Luster M. Dermal phospho-alpha-synuclein deposits confirm REM sleep behaviour disorder as prodromal Parkinson's disease. Acta Neuropathol. (2017) 133:535–45. 10.1007/s00401-017-1684-z 28180961 PMC5348554

[66] Siepmann T Illigens BM-W Barlinn K. Alpha-synuclein in cutaneous small nerve fibers. Neuropsychiatr Dis Treat. (2016) 12:2731–5. 10.2147/NDT.S117423 27822045 PMC5087811

[67] Vinik AI Erbas T Casellini CM. Diabetic cardiac autonomic neuropathy, inflammation and cardiovascular disease. J Diabetes Investig. (2013) 4:4–18. 10.1111/jdi.12042 23550085 PMC3580884

[68] Freeman R. Diabetic autonomic neuropathy. Handbook Clin. Neurol. (2014) 126:63–79. 10.1016/B978-0-444-53480-4.00006-0 25410215

[69] Pop-Busui R Evans GW Gerstein HC Fonseca V Fleg JL Hoogwerf BJ. Effects of Cardiac Autonomic Dysfunction on Mortality Risk in the Action to Control Cardiovascular Risk in Diabetes (ACCORD) Trial. Diabetes Care. (2010) 33:1578–84. 10.2337/dc10-0125 20215456 PMC2890362

[70] Verrotti A Prezioso G Scattoni R Chiarelli F. Autonomic neuropathy in diabetes mellitus. Front Endocrinol. (2014) 5:205. 10.3389/fendo.2014.00205 25520703 PMC4249492

[71] Rolim LC de Souza JST Dib SA. Tests for early diagnosis of cardiovascular autonomic neuropathy: critical analysis and relevance. Front Endocrinol. (2013) 4:173. 10.3389/fendo.2013.00173 24273533 PMC3822331

[72] Krishnan ST Rayman G. The LDIflare: a novel test of C-fiber function demonstrates early neuropathy in type 2 diabetes. Diabetes Care. (2004) 27:2930–5. 10.2337/diacare.27.12.2930 15562209

[73] Callaghan BC Xia R Reynolds E Banerjee M Rothberg AE Burant CF. Association between metabolic syndrome components and polyneuropathy in an obese population. JAMA Neurol. (2016) 73:1468–76. 10.1001/jamaneurol.2016.3745 27802497 PMC5217829

